# Spatiotemporal monitoring and assessment of green oak leaf-roller outbreaks in Zagros forests using Sentinel-2 data and the BFAST algorithm

**DOI:** 10.1038/s41598-026-48040-1

**Published:** 2026-04-20

**Authors:** Hadi Beygi Heidarlou, Stelian Alexandru Borz

**Affiliations:** 1 Forestry Department, Faculty of Natural Resources, Urmia University, P.O. Box 165, Urmia, Iran; 2 Department of Forest Engineering, Forest Management Planning and Terrestrial Measurements, Faculty of Silviculture and Forest Engineering, Transilvania University of Brașov, 500123 Brașov, Romania

**Keywords:** Adaptive pest management, Forest health monitoring, Pest impacts, Remote sensing, Time series analysis, Vegetation indices, Zagros oak forests, Ecology, Ecology, Environmental sciences

## Abstract

The decline of oak forests in the Zagros Mountains poses a major threat to the ecological stability and long-term resilience of these ecosystems. This study quantified the effects of green oak leaf-roller (*Tortrix viridana* L.) outbreaks on canopy dynamics by integrating Sentinel-2 imagery, vegetation indices (NDVI, EVI, NDWI), and the BFAST time-series algorithm. Canopy deterioration was most pronounced at mid-elevations (1200–1800 m) and on moderately sloped terrain (15–30%). The most severe defoliation occurred in 2019 (~ 4253 ha), coinciding with the peak infestation period, followed by partial stabilization in 2021 (~ 1362 ha) and 2022 (~ 1380 ha). Vegetation indices revealed marked physiological stress in affected stands, with NDVI declining to ~ 0.38, EVI to ~ 0.23, and NDWI to ~ 0.1, indicating reductions in photosynthetic activity, canopy density, and crown moisture. Although partial recovery was observed in 2023–2024, persistent biotic pressure limited full canopy regeneration. Time-series modeling showed that spatial and temporal variation in impacted stands was jointly shaped by topography, distance to roads and rivers, drought intensity, and pest activity. The combined use of high-resolution satellite data and BFAST provided an effective framework for detecting subtle canopy degradation and supports more accurate monitoring and adaptive management of pest-induced forest decline.

## Introduction

Forests, as fundamental components of terrestrial ecosystems, play a vital role in maintaining ecological balance. They regulate energy and biogeochemical cycles, sequester carbon, produce oxygen, moderate local climate, protect soil and water resources, and support biodiversity, making them among the most critical natural assets on the planet^1^. Globally, forests cover approximately one-third of the Earth’s land surface and provide habitat for over 80% of terrestrial species^2^.

In Iran, the Zagros region holds particular ecological significance, stretching from the northwest to the southwest of the country and accounting for roughly 40% of the nation’s total forested area. Dominated by various oak species (*Quercus* spp.), these forests provide essential ecological functions while supporting the livelihoods, food security, and cultural sustainability of local communities^3^.

The concept of “forest health” is a key indicator in sustainable natural resource management, reflecting the biological, physiological, and structural condition of forests, as well as their ability to maintain ecosystem functions under environmental stress^4^. Forest health is assessed through multiple parameters, including species density and diversity, canopy greenness, leaf loss and dieback, soil quality, and photosynthetic capacity^5^. Monitoring forest health in vulnerable regions, such as the Zagros, is crucial because decline in tree vigor can trigger cascading ecological consequences, including reduced carbon storage, habitat degradation, and gradual ecosystem decline^6^.

In recent decades, Zagros forests have experienced concurrent pressures from climate change, recurrent droughts, widespread wildfires, overgrazing, land-use change, and outbreaks of pests and diseases^7^. The cumulative effects of these stressors can lead to reduced forest vitality, characterized by gradual decreases in growth, canopy condition, and ecological functionality, which may ultimately increase the risk of tree mortality if unfavorable conditions persist^8^.

Among the most significant biotic threats is the oak leaf-roller moth (*Tortrix viridana* L.), a species native to Mediterranean regions, North Africa, Western Asia, and parts of Europe^9^. In Iran, this pest has become increasingly widespread, particularly in West Azerbaijan, Fars, and Lorestan provinces. The moth feeds on oak buds and young leaves, substantially reducing the photosynthetically active surface area and disrupting tree growth and physiological balance^10^. In certain hotspots, over 50% of oak canopy has been reported to suffer complete defoliation^11^. In the Zagros, *T. viridana* completes one generation per year, passing through five larval instars. Larval activity begins from late March to April, coinciding with budburst, and mature larvae pupate by May. Adult moths emerge from late May to early June, with oviposition continuing through June and July^12^.

The ecological consequences of leaf-roller outbreaks extend beyond immediate growth reductions. They affect carbon cycling, increase vulnerability to drought and heat stress, degrade water resources, accelerate soil erosion, and threaten biodiversity^13^. Climate change can exacerbate these impacts through synergistic interactions between thermal–hydric stress and biotic pressures, further accelerating forest decline^14^. Beyond the overall presence of the pest, the spatial patterns and intensity of these outbreaks are often influenced by topographic characteristics such as elevation and aspect^15^. These factors modulate the local microclimate, which affects the phenological synchronization between oak budburst and larval emergence^16^. Understanding how topography shapes the severity of defoliation is essential for predicting which forest stands are most vulnerable to long-term decline.

Accurate and systematic monitoring of forest health is therefore indispensable. Traditional field-based methods offer high precision but are limited in spatial coverage, time-consuming, and costly. In contrast, remote sensing technologies, particularly multispectral satellite data such as Sentinel-2, provide a powerful tool for large-scale forest monitoring^17^. Sentinel-2 imagery, with a 10-m spatial resolution, 13 spectral bands, and a 5-day revisit cycle, enables detailed assessment of temporal and spatial variations in canopy greenness and forest condition^18^.

Vegetation indices such as Normalized Difference Vegetation Index (NDVI), Normalized Difference Water Index (NDWI), and Enhanced Vegetation Index (EVI) have been widely used to quantify physiological changes and detect biotic stressors, including pest outbreaks^19^. Furthermore, the BFAST (Breaks For Additive Seasonal and Trend) algorithm, developed by Verbesselt, et al. ^20^, has emerged as an effective tool for time-series analysis of remote sensing data. By decomposing seasonal and trend components and identifying breakpoints, BFAST can detect both abrupt disturbances (e.g., pest outbreaks) and gradual changes (e.g., drought), offering precise insights into temporal dynamics^21^. Crucially, the magnitude of these detected breakpoints can serve as a proxy for pest intensity, allowing for a more nuanced assessment of forest degradation beyond simple presence/absence detection.

Despite these methodological advances, comprehensive studies on monitoring oak leaf-roller outbreaks using remote sensing in Iran remain limited, especially at regional scales. Most domestic studies have focused on general forest decline, rarely examining the spatial–temporal dynamics of pest impacts. Furthermore, the role of site-specific factors, particularly topographic characteristics such as elevation, slope, and aspect, in shaping the vulnerability of oak stands to infestation remains poorly understood in the Zagros context. Topography significantly influences microclimate and phenological synchronization between the host and the pest^22^, yet its relationship with outbreak patterns is often overlooked. This gap limits understanding of fine-scale distribution, temporal progression, and ecological consequences of outbreaks in the Zagros. Integrating Sentinel-2 imagery, vegetation indices, and time-series analysis presents a promising approach to address this knowledge gap and provides a robust foundation for continuous forest health monitoring.

Accordingly, the primary objective of this study was to assess the impact of oak leaf-roller moth outbreaks on the health of Zagros forests in southern West Azerbaijan Province. We employed multispectral Sentinel-2 data, vegetation indices, and the BFAST algorithm to analyze temporal and spatial trends in canopy greenness and identify affected areas. Additionally, the study examined the relationships between canopy health variation and topographic factors such as elevation, slope, and aspect, providing a scientific basis for mapping pest distribution and informing management strategies to mitigate pest impacts and enhance ecosystem resilience.

Specifically, this research addresses the following questions:

1. Can Sentinel-2 satellite imagery reliably detect and distinguish oak trees affected by the oak leaf-roller moth from healthy trees?
2. Can the BFAST algorithm accurately identify temporal breakpoints caused by pest outbreaks and generate precise maps of affected areas?
3. How do topographic characteristics influence the spatial patterns and intensity of pest infestation?

## Results

### Temporal dynamics of NDVI (2018–2024)

**Fig. 1:**
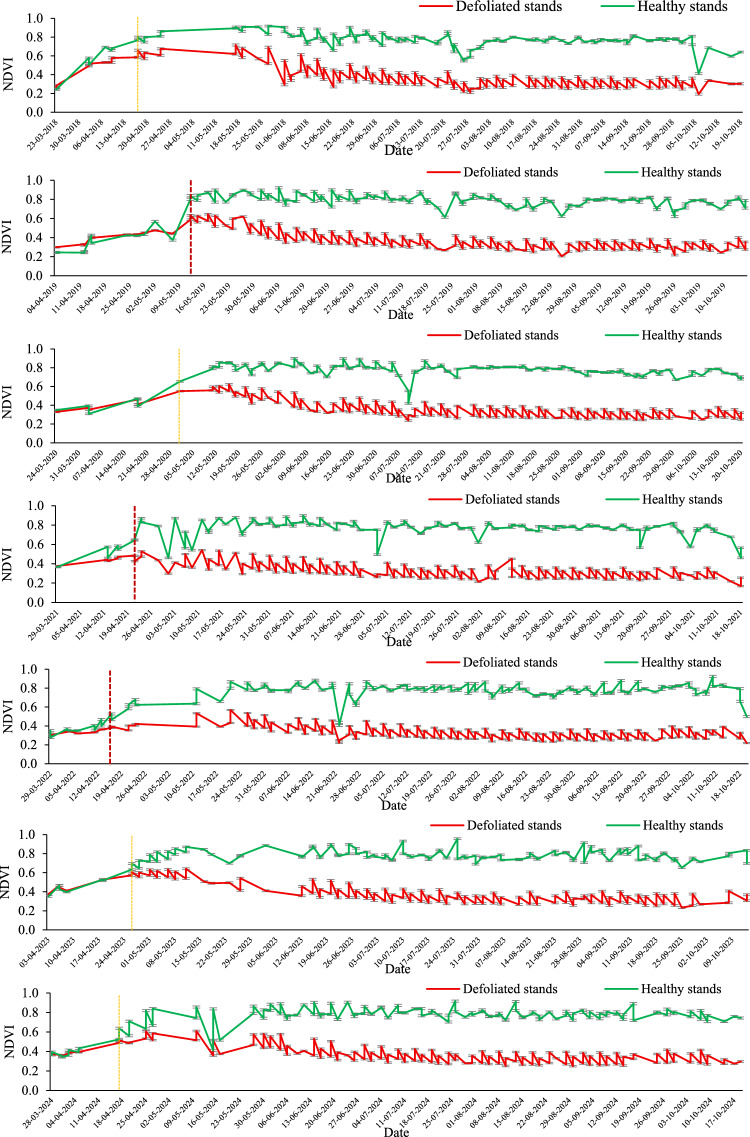
NDVI trends in healthy and defoliated (affected) oak stands (2018–2024) with BFAST breakpoints; error bars represent the standard error (± 1 SE).

The results obtained from the BFAST model, applied to Sentinel-2 time-series data from 2018 to 2024, indicated that the NDVI followed a similar seasonal pattern in both canopy types (affected and non-affected) (Fig. 1). However, the model successfully identified structural breakpoints in the NDVI time series, enabling precise differentiation between healthy and affected areas.

The model detected three major breakpoints in 2019, 2021, and 2022. The first breakpoint occurred in the first half of 2019, coinciding with a decline in NDVI in portions of the affected stands, suggesting the onset of canopy stress in these areas. The second breakpoint appeared around mid-2021, reflecting a period during which NDVI values remained relatively stable, suggesting no additional changes in canopy conditions. In contrast, the third breakpoint, observed in summer 2022, revealed a significant NDVI reduction associated with pronounced canopy stress, which coincided with the intensification of drought and environmental stressors.

At the beginning of the study period (2018), the mean NDVI was 0.80 in healthy stands and 0.55 in affected canopy areas. Between 2019 and 2020, NDVI increased in both canopy types (reaching approximately 0.85 in healthy stands and 0.60 in affected stands at the peak of the growing season, May to June), although fluctuations in the trend were evident in the affected stands. In 2021, NDVI changes were limited and relatively stable across both canopy types. In 2022, BFAST identified a sudden and significant decline in NDVI in the affected stands, with mean values dropping from ~ 0.60 to 0.38, whereas healthy stands experienced only a modest decrease from 0.78 to 0.62. During 2023 and 2024, a relative recovery in NDVI was observed in both canopy types, although the BFAST outputs still captured unstable seasonal fluctuations in the affected stands. By 2024, mean NDVI remained around 0.80 in healthy stands and between 0.50 and 0.55 in the affected canopy areas.

Analysis of the seasonal component revealed a three-stage pattern in the annual NDVI cycle for both canopy types. NDVI values increased from late March to May, reached a maximum during late May and June, and subsequently declined from July to September. However, the model outputs showed a reduction in seasonal amplitude for the affected stands, where a premature decline in NDVI values was observed. This shift effectively truncates the natural peak of the growing season in infested areas, with peak NDVI occurring approximately 2–3 weeks earlier compared to healthy stands.

### Temporal dynamics of EVI (2018–2024)

**Fig. 2:**
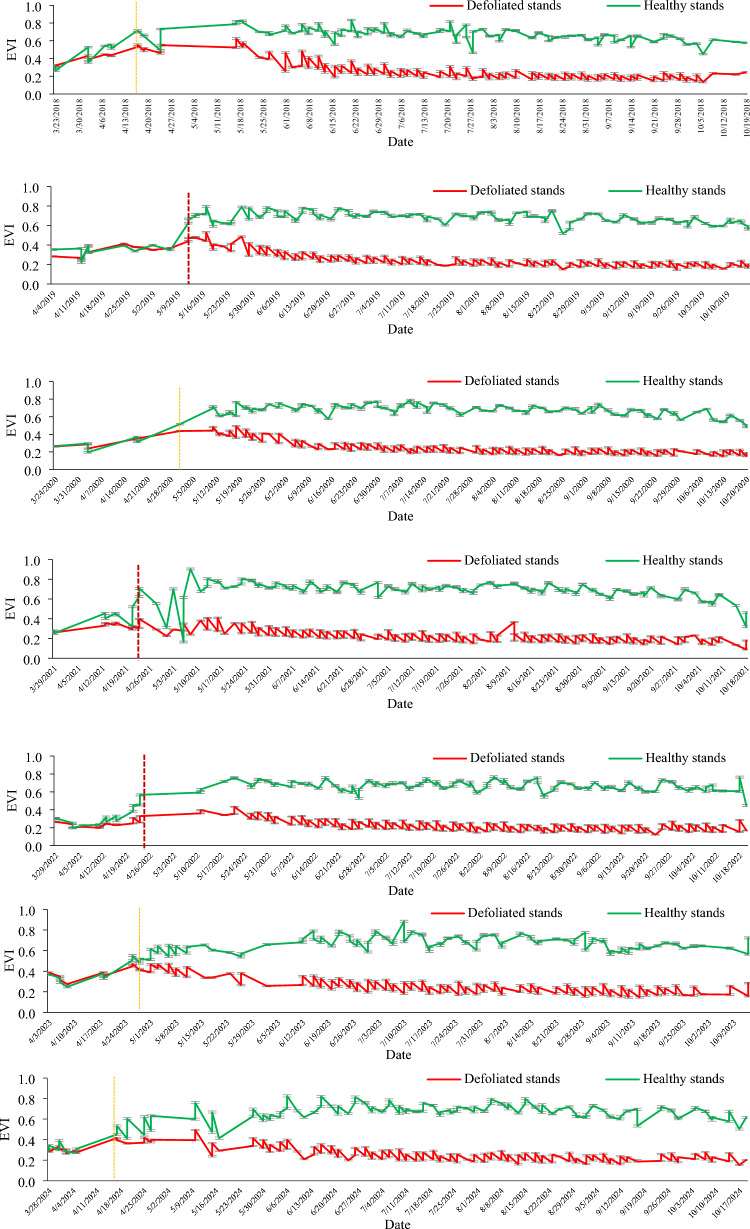
EVI trends in healthy and defoliated (affected) oak stands (2018–2024) with BFAST breakpoints; error bars represent the standard error (± 1 SE).

Analysis of EVI temporal trends using the BFAST model indicated that both affected and healthy canopies exhibited similar seasonal patterns (Fig. 2). Nevertheless, the amplitude of variation and trend stability were considerably lower in the affected canopies. The BFAST model identified three notable breakpoints in the EVI time series, occurring in 2019, 2021, and 2022.

The first breakpoint, identified in early 2019, coincided with a decline in EVI across the affected tands. The second breakpoint, detected around mid-2021, reflected temporary stability and partial recovery in both canopy types. The third breakpoint, in summer 2022, corresponded to a sharp structural decline in EVI within affected stands, overlapping with recorded drought periods.

At the beginning of the study (2018), mean EVI values were about 0.65 for healthy stands and 0.27 for affected ones. Between 2019 and 2020, the model recorded a relative increase in EVI for both canopy types (reaching about 0.70 and 0.35, respectively, during the peak growing season, May to June), although fluctuations were greater in affected stands. In 2021, EVI remained relatively stable in both canopy types, whereas in 2022, a noticeable decline was observed in the affected stands, with mean EVI decreasing from 0.33 to 0.23, compared to a decrease from 0.68 to 0.57 in healthy stands. In 2023–2024, a slight recovery trend appeared in both canopy types, although the model indicated more irregular seasonal oscillations and a narrower range of variation in the affected stands. By the end of the study period, mean EVI stabilized around 0.65 in healthy stands and between 0.30 and 0.33 in impacted stands.

Analysis of the seasonal component extracted from the model indicated that the annual EVI cycle in both canopy types followed a similar three-phase trajectory. At the onset of the growing season, EVI increased from late March to May, coinciding with budburst and leaf expansion. From late May to June, EVI reached its annual maximum, reflecting peak canopy greenness, and then gradually declined from July to September, corresponding to the end of active growth and reduced soil moisture.

A comparative assessment showed that the seasonal amplitude of EVI in affected stands was, on average, 20–30% lower than in healthy stands, and the timing of the photosynthetic peak occurred roughly two weeks earlier. These patterns indicate shorter and less stable seasonal growth dynamics in the affected stands.

### Temporal dynamics of NDWI (2018–2024)

**Fig. 3:**
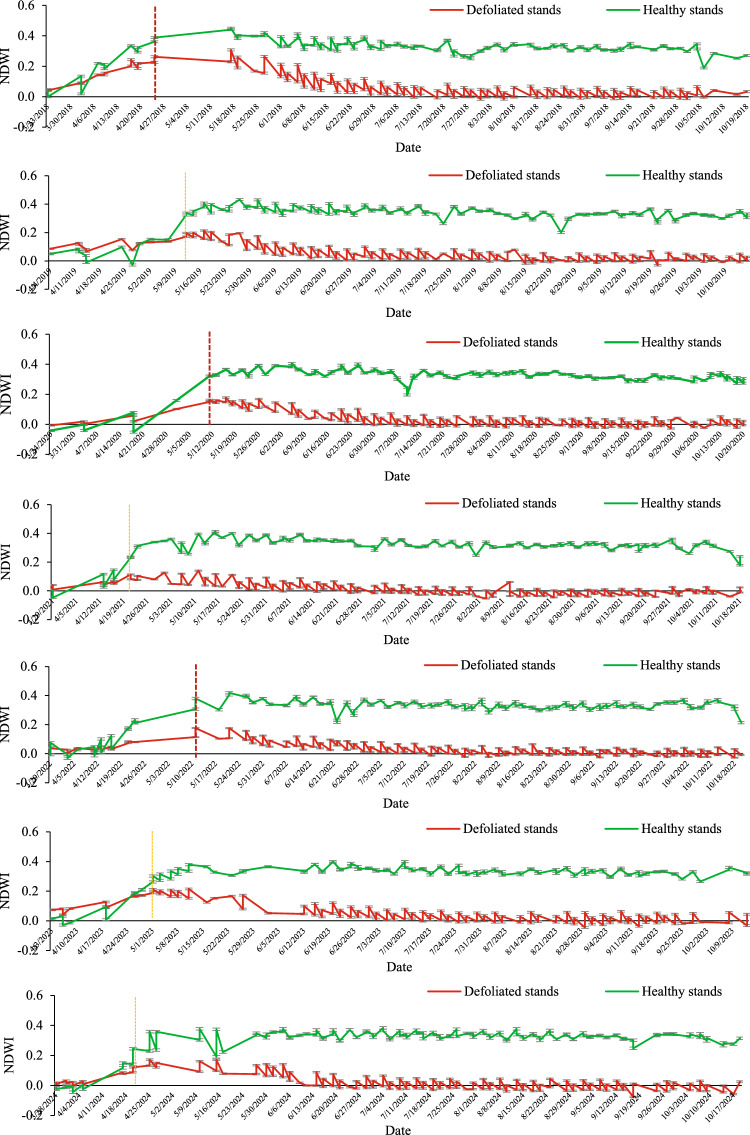
NDWI trends in healthy and defoliated (affected) oak stands (2018–2024) with BFAST breakpoints; error bars represent the standard error (± 1 SE).

The BFAST model results for NDWI showed that changes in this index followed a similar seasonal pattern in both affected and healthy canopies (Fig. 3). However, the seasonal amplitude and overall trend stability of NDWI were considerably lower in the affected canopies than in the healthy ones. This suppressed amplitude reflects the inability of defoliated stands to reach normal canopy water-retention levels during the peak growing season, a pattern that consistently mirrors the EVI dynamics described above.

The BFAST model identified three structural breakpoints in the NDWI time series. The first breakpoint occurred in the first half of 2018, coinciding with a pronounced decrease in NDWI in the defoliated stands. The second breakpoint was observed in mid-2020 and corresponded to a general decline in NDWI across both canopy types, with a more substantial decrease in the defoliated stands. The third breakpoint occurred in early summer 2022, reflecting a sharp decline in NDWI within the defoliated stands.

At the beginning of the study period, mean NDWI values were estimated at approximately 0.35–0.40 in healthy stands and 0.0–0.2 in defoliated stands. During 2019 and 2020, a relative increase in NDWI was observed in both groups, although the defoliated stands exhibited greater variability. From 2021 to 2023, a gradual decline in NDWI persisted in the defoliated stands, whereas values in healthy stands remained relatively stable at around 0.3–0.4. In 2024, NDWI continued to decrease slightly in defoliated stands, while changes in healthy stands remained comparatively stable.

Analysis of the seasonal component extracted from the model revealed that the annual NDWI cycle in both canopy types followed a similar three-phase pattern. At the beginning of the growing season, NDWI increased from late March to May. During the peak growing season, NDWI in healthy stands reached its maximum (0.35–0.40), whereas in defoliated stands it remained limited to 0.1–0.2. Towards the end of the growing season, NDWI gradually declined from July to September. On average, the seasonal amplitude of NDWI in defoliated stands was 25–30% lower than in healthy stands, and the timing of the NDWI peak occurred approximately one to two weeks earlier.

### Analysis of climatic fluctuations (SPEI) and vegetation responses

**Fig. 4:**
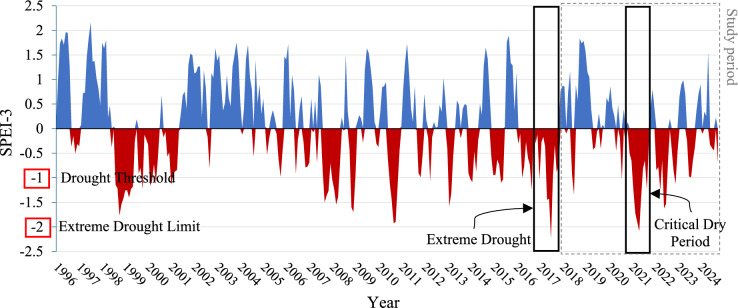
Temporal variations of the 3-month Standardized Precipitation Evapotranspiration Index (SPEI-3) in study area from 1996 to 2024.

The analysis of the three-month Standardized Precipitation Evapotranspiration Index (SPEI-3) for study area (i.e., Sardasht County) from 1996 to 2024 reveals significant climatic fluctuations that have profoundly impacted the health of the region’s oak forests (Fig. 4). The results indicate that Sardasht experienced successive moderate to severe droughts during the late 1990s and early 2000s, particularly between 1999 and 2001. Following a relatively humid period in the mid-2000s, the region entered a new phase of intense climatic variability. Notably, the most severe climatic stress in the past 30 years occurred in 2017, with SPEI values plummeting to approximately − 2.21, representing an *extreme drought*. This extreme drought may have increased the vulnerability of oak trees to subsequent pest outbreaks.

Throughout the primary study period (2018–2024), the climatic conditions in study area exhibited distinct fluctuations that directly influenced forest canopy dynamics. Following the extreme drought of 2017, the 2018–2019 period was characterized by a notable recovery, with SPEI values reaching + 1.78, indicating a shift toward humid conditions. However, despite this marked moisture improvement, NDVI and EVI continued to show declining trends in several forest patches, suggesting a lack of immediate synchronization between climatic recovery and vegetation vigor.

This recovery phase was followed by a critical moisture deficit in 2021, where SPEI values dropped to − 2.08. This event was recorded as the second-most severe drought in the region’s 30-year history. During this crisis, a clear synchrony was observed between climatic stress and a sharp decline in canopy indices. Subsequently, the period of 2023–2024 showed a return to more favorable conditions; while 2023 was marked by sinusoidal moisture fluctuations, 2024 experienced a significant recovery with positive SPEI values reaching + 1.52 during the peak growing season.

### Temporal dynamics of canopy changes induced by oak leaf-roller outbreaks (2018–2024)

**Fig. 5:**
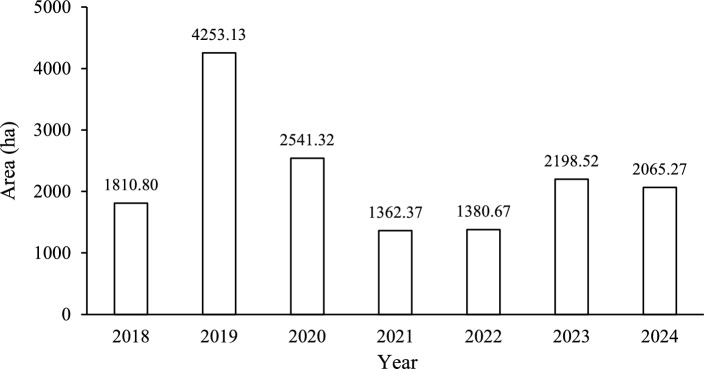
Temporal trends of areas affected by pest outbreaks (2018–2024) based on BFAST analysis.

**Fig. 6:**
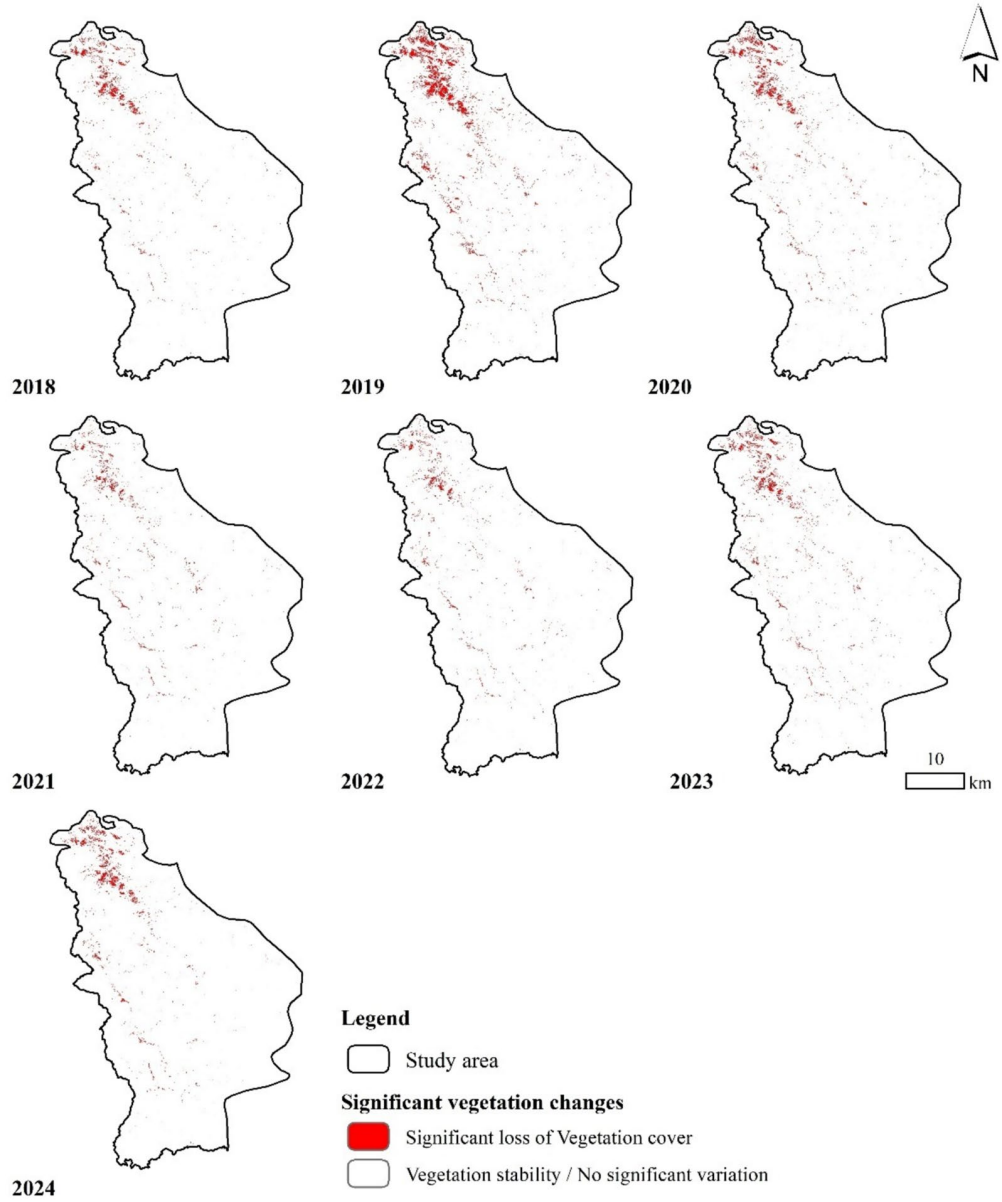
Spatial pattern of significant vegetation changes (2018–2024) derived from the BFAST model.

Using the BFAST model, along with field-verified ground truth data and Sentinel-2 imagery analysis, substantial temporal fluctuations were observed in areas showing significant vegetation decline between 2018 and 2024 (Figs. 5 and 6). In 2018, approximately 1810.80 hectares of the surveyed forests were affected by pest outbreaks, representing an initial point for detecting notable vegetation changes. In 2019, the area affected by the pest outbreak increased sharply to 4253.13 hectares, representing the peak extent observed during the study period and reflecting a substantial impact on forest canopy conditions. This surge may be attributed to favorable climatic conditions for pest population growth (e.g., drought or elevated temperatures), reduced resistance of host trees, or a combination of these factors.

In 2020, the affected area decreased to 2541.32 hectares, which, although lower than the previous year, remained higher than in 2018. This reduction may reflect a relative alleviation of pest pressure or the initiation of partial canopy recovery. The lowest changes within the study period were observed in 2021 (1362.37 hectares) and 2022 (1380.67 hectares).

From 2023 onwards, an upward trend in vegetation changes was observed again, with the affected area reaching 2198.52 hectares in 2023 and 2065.27 hectares in 2024. Although the outbreak intensity did not reach the peak levels of 2019, these values indicate that pest activity continues to pose a threat to the sustainability of forest vegetation in the region.

**Fig. 7:**
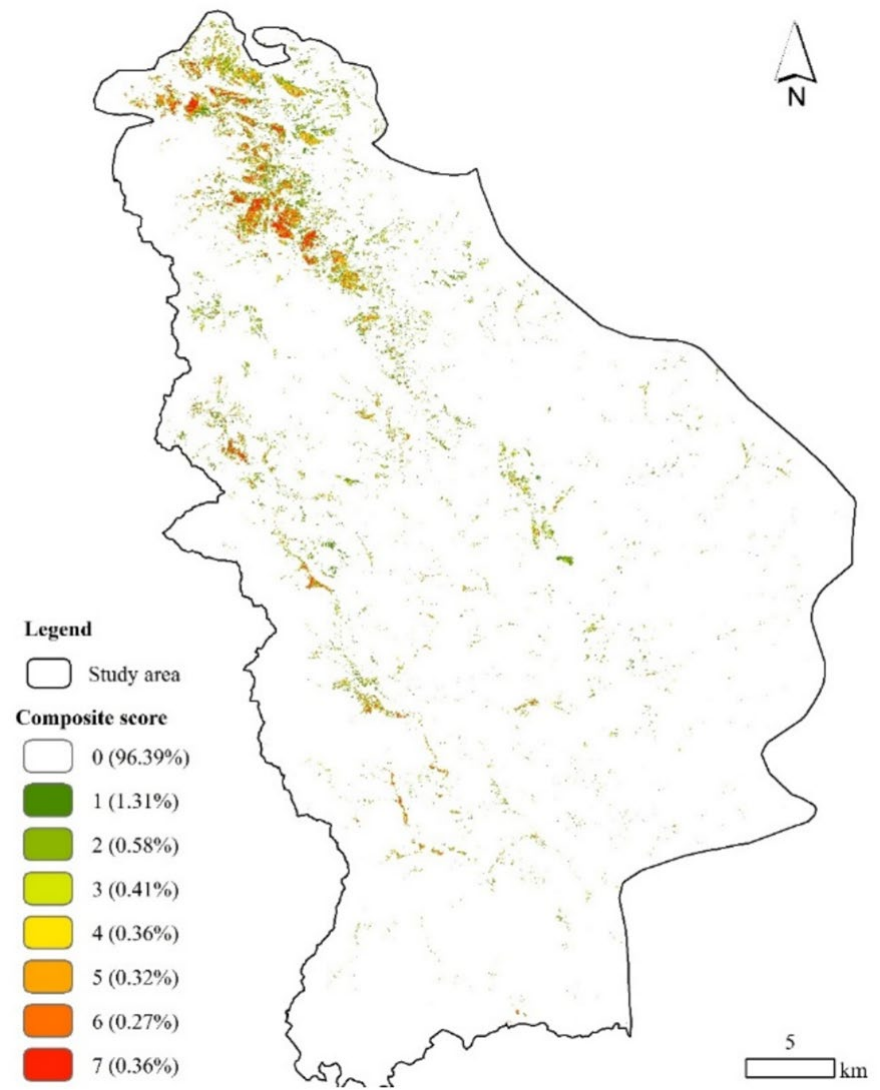
Spatial distribution of areas experiencing recurring vegetation changes (2018–2024) derived from combined annual maps.

The combined analysis of seven annual vegetation maps enabled the identification of areas that experienced recurring vegetation changes over the study period, specifically associated with oak leaf-roller outbreaks. A small fraction of the area underwent repeated changes: 1.31% over six years, 0.58% over five years, 0.41% over four years, 0.36% over three years, 0.32% over two years, 0.27% over one year, and 0.36% over all seven years (Fig. 7). The spatial distribution of these classes depicts areas with varying frequencies of vegetation change, ranging from highly stable regions (class 0) to areas affected in multiple years (classes 1–7).

### Accuracy assessment

The reliability of the satellite-derived findings was validated against 100 ground-truth points across the entire study period. Table 1 details the cross-tabulation of observed versus predicted vegetation states, while Table 2 summarizes the annual accuracy metrics. The classification achieved a high level of performance, with Overall Accuracy (OA) ranging from 94 to 97% (mean = 95.3%) and Kappa coefficients varying between 0.88 and 0.94. Specifically, the Producer’s accuracy averaged 96.1% for the healthy class and 93.4% for the defoliated class, while User’s accuracy reached 96.3% and 94.6%, respectively. Analysis of classification errors revealed that Omission errors (false negatives) for healthy regions remained low, ranging from 1.67 to 6.67%, while defoliated regions exhibited Omission errors between 3.28 and 7.5%. Similarly, Commission errors (false positives) fluctuated between 4.92 and 7.5% for healthy regions and 2.56–9.52% for defoliated regions. These results indicate consistent model performance across the study years and confirm the robustness of the dataset for analyzing long-term vegetation change trends.

**Table 1:** Confusion matrix of the vegetation classification (Healthy vs. Defoliated regions) based on ground-truth points for the period 2018–2024.

		Predicted													
		Healthy region							Defoliated region						
Study years		2018	2019	2020	2021	2022	2023	2024	2018	2019	2020	2021	2022	2023	2024
Reference data	Healthy region	58	57	56	59	57	58	59	2	3	4	1	3	2	1
	Defoliated region	3	2	2	2	3	3	2	37	38	38	38	37	37	38

**Table 2:** Accuracy assessment of BFAST-derived vegetation changes results for 2018–2024.

Index		2018	2019	2020	2021	2022	2023	2024
Overall Accuracy (%)		95	95	94	97	94	95	97
Kappa Coefficient (%)		0.90	0.90	0.88	0.94	0.88	0.90	0.94
Producer’s Accuracy (%)	Healthy region	96.7	95.0	93.3	98.3	95.0	96.7	98.3
	Defoliated region	92.5	95.0	95.0	95.0	92.5	92.5	95.0
User’s Accuracy (%)	Healthy region	95.1	96.6	96.5	96.7	95.0	95.1	96.7
	Defoliated region	94.9	92.7	90.5	97.4	92.5	94.9	97.4
Omission (False Negative) (%)	Healthy region	3.33	5.00	6.67	1.67	5.00	3.33	1.67
	Defoliated region	7.50	3.39	3.45	3.28	5.00	4.92	3.28
Commission (False Positive) (%)	Healthy region	4.92	5.00	5.00	5.00	7.50	7.50	5.00
	Defoliated region	5.13	7.32	9.52	2.56	7.50	5.13	2.56

### Statistical analysis of spatial distribution of affected patches

Chi-square Goodness of Fit tests were conducted to examine the distribution of outbreak patches across topographic and spatial variables, including elevation, slope, aspect, distance to roads, and distance to rivers (Table 3).

**Table 3:** Chi-square test results for patch distribution across environmental variable classes.

Variable	Classes	Observed N	Expected N	Residual	Chi-Square	df	p-value
Elevation (m)	< 1200	11	66.7	− 55.7	304.69	2	< 0.001
	1200–1800	183	66.7	116.3			
	> 1800	6	66.7	− 60.7			
Slope (%)	0–15	27	66.7	− 39.7	43.57	2	< 0.001
	15–30	70	66.7	3.3			
	> 30	103	66.7	36.3			
Aspect	Flat	5	40	− 35.0	102.4	4	< 0.001
	North	81	40	41.0			
	East	66	40	26.0			
	South	23	40	− 17.0			
	West	25	40	− 15.0			
Distance from road (m)	0–500	99	66.7	32.3	30.73	2	< 0.001
	500–1000	66	66.7	− 0.7			
	> 1000	35	66.7	− 31.7			
Distance from river (m)	0–200	47	66.7	− 19.7	70.81	2	< 0.001
	200–500	31	66.7	− 35.7			
	> 500	122	66.7	55.3			

Patch distribution across elevation classes differed significantly from uniformity (χ^2^ = 304.69, df = 2, *p* < 0.001), with the highest density in the mid-elevation class (1200–1800 m) and lower densities in the lower and upper classes. For slope, patches were predominantly located on steep slopes (> 30%), showing a significant deviation from uniform distribution (χ^2^ = 43.57, df = 2, *p* < 0.001). Aspect also influenced patch occurrence (χ^2^ = 102.40, df = 4, *p* < 0.001), with the highest density in north-facing aspects and lower densities in other directions. Proximity to roads significantly affected patch distribution (χ^2^ = 30.73, df = 2, *p* < 0.001), with the greatest density in areas within 0–500 m of roads. Similarly, distance to rivers was a significant factor (χ^2^ = 35.78, df = 2, *p* < 0.001), with patches concentrated beyond 500 m and lower densities at closer distances.

## Discussion

The findings of this study revealed that the spatiotemporal dynamics of oak-dominated forests in southern West Azerbaijan Province were strongly influenced by outbreaks of the oak leaf-roller (*T. viridana* L.). Time-series analysis using Sentinel-2-derived NDVI, EVI, and NDWI, coupled with BFAST breakpoint detection, indicated that the highest intensity of forest decline occurred at mid-elevations and on moderately sloped terrains, highlighting the heightened susceptibility of the Zagros mountain ecosystems to pest disturbances.

These results are consistent with previous studies in the Zagros region. Hosseinzadeh, et al.^23^ in Ilam, western Iran, demonstrated that topographic variables, including elevation, slope, and aspect, play a pivotal role in determining pest-induced forest decline. Similarly, Shiravand, et al.^24^, using BFAST for monitoring Zagros forests, reported that a decline in oak forests induced the greatest vegetation loss. Therefore, the vulnerability of Zagros oak forests to insect pests is influenced not only by the biological traits of oak species but also by environmental factors.

International comparisons show similar patterns. Lambert, et al.^25^ in Canada and Ibañez-Álvarez, et al.^26^ in Spain reported that pest outbreak cycles are consistently associated with marked reductions in vegetation indices, with more pronounced effects in mountainous regions with variable moisture conditions. Moreover, Wu, et al.^21^ highlighted that drought and climatic fluctuations exacerbate pest outbreaks, accelerating forest decline. Our findings corroborate these observations, as severe reductions in NDVI, EVI, and NDWI were recorded during 2019 and 2022. The SPEI-3 analysis confirms that these biotic crises were preceded by extreme climatic stress; specifically, the extreme drought of 2017 (SPEI ≈ − 2.21) acted as a predisposing factor for the 2019 outbreak, while the severe moisture deficit in 2021 (SPEI ≈ − 2.08) exacerbated the 2022 infestation. These results suggest a clear temporal link where multi-year drought peaks identified by SPEI dictate the subsequent intensity of pest-induced canopy loss, consistent with the host-stress hypothesis^27^.

Analysis of vegetation indices indicated that significant reductions in canopy photosynthetic activity and density occurred in 2022. In defoliated stands, NDVI decreased from ~ 0.60 to 0.38, EVI dropped from 0.33 to 0.23, and NDWI ranged from 0.2 to 0.1. Conversely, healthy stands exhibited smaller changes, with NDVI decreasing from 0.78 to 0.62, EVI from 0.68 to 0.57, and NDWI remaining relatively stable at 0.35–0.40.

Notably, some affected stands exhibited lower baseline vegetation indices even before detected breakpoints. This pre-existing discrepancy likely reflects cumulative structural and physiological degradation, such as reduced leaf area index (LAI), crown thinning, and altered canopy architecture from prior defoliation cycles^28^. Additionally, increased soil background reflectance in open canopies and site-specific stressors (e.g., drought or edaphic constraints) may further suppress spectral greenness^29^. Despite selecting homogeneous oak cover to minimize mixed-pixel effects, inherent variations in stand density and vertical complexity remain difficult to fully quantify via satellite data. Consequently, these baseline differences, partly unrelated to specific outbreak events, may influence subsequent seasonal trajectories^30^.

While background soil and shadowing inherently lower the absolute index values in sparse oak stands, the BFAST model focuses on the relative magnitude of change rather than absolute baselines^20^. The abrupt drop-and-recovery pattern, synchronized with the larval feeding window, distinguishes pest-induced defoliation from permanent structural characteristics across all stand densities. Furthermore, our analysis confirmed that defoliated pixels exhibited a significant reduction in seasonal amplitude and a 2–3 week advancement in peak photosynthetic timing compared to healthy stands. This temporal shift, combined with the magnitude of decline, ensures that the detected anomalies are driven by rapid leaf area loss regardless of initial canopy density.

The premature decline in NDVI and the truncation of the peak greenness period serve as direct indicators of canopy trauma. This phenological shift is driven by rapid defoliation during the *T. viridana* larval peak, which physically disrupts the normal physiological development and seasonal trajectory of the oak stands. This advancement of the peak is an artifact of rapid canopy loss rather than a true phenological shift. While both drought and pest outbreaks reduce vegetation indices, their temporal signatures differ significantly. Unlike the gradual, widespread decline typical of drought, the BFAST model identified abrupt, localized breakpoints during the spring of 2022, precisely aligning with the *T. viridana* larval feeding window^31^. Crucially, while SPEI data for 2022–2024 (reaching + 1.52) indicate a shift toward favorable moisture conditions, persistent negative NDVI and NDWI anomalies confirm a biotic driver. This ‘decoupling’ of vegetation greenness from climatic recovery suggests that once a pest-driven degradation threshold is reached, improved precipitation is insufficient for immediate canopy restoration^32^. This is most evident in 2024, where canopy signals remained significantly below the ecological baseline despite peak moisture recovery, further validating the dominance of pest impact over climatic factors. Our 2022 field surveys confirmed high larval densities and extensive canopy stripping. This field-based evidence allowed us to decouple the spectral signals, attributing the sharp NDVI and NDWI drops to the synergistic impact of pest outbreaks exacerbated by water stress, rather than moisture deficit alone.

Seasonal observation frequency was constrained by atmospheric and topographic factors. In the Zagros region, persistent cloud cover, topographic shadowing, and early-spring snow limited the availability of valid Sentinel-2 observations before full leaf development^33^. Since the BFAST algorithm characterizes structural changes relative to each pixel’s historical baseline, data sparsity during this critical phenological period may introduce uncertainties in the timing (lead/lag effects) and magnitude of detected breakpoints. Consequently, early-stage larval defoliation signals may exhibit inherent temporal latency or reduced spectral sensitivity^34^.

Furthermore, the distinction between healthy and infested stands lies in the residual anomalies and the timing of the decline, rather than overall seasonal synchronization. While healthy stands follow predictable phenological patterns tied to regional climatic stress, infested stands exhibit an additional, abrupt spectral dip captured by BFAST that exceeds normal variability. Although the magnitude difference may be subtle, the statistical significance of these breakpoints during peak larval activity identifies a distinct departure from the expected phenological curve^35^. This decoupling from the regional greenness trend provides the ecological evidence needed to specifically attribute these changes to *T. viridana* defoliation.

Temporal analysis of the area affected by significant vegetation decline revealed substantial fluctuations. In 2018, approximately 1810.8 ha were impacted, representing initial pest-induced changes. The largest extent occurred in 2019 (~ 4253 ha), indicating peak outbreak intensity. The 2019 peak was validated against high-resolution imagery and official pest records, confirming *T. viridana* as the primary driver. Unlike wildfires, which yield near-zero index values and burn scars, or droughts, which manifest as gradual, regional declines, the 2019 signal exhibited the abrupt defoliation-refoliation cycle characteristic of oak leaf-roller larvae^36^.

Affected stands transitioned from high intra-annual variability in 2019–2020 to a narrower seasonal range by 2023–2024. Early in the outbreak, marked defoliation-refoliation cycles, driven by secondary shoots (lammas growth) following initial canopy loss, resulted in significant spectral fluctuations^16^. Conversely, repeated stress likely exhausted non-structural carbohydrate (NSC) reserves in later years, diminishing regenerative capacity and producing a flattened phenological curve with reduced amplitude^37^. This pattern underscores the progressive physiological impact of recurring *T. viridana* outbreaks^28^, leading to chronic suppression of oak canopy vigor.

In 2020, affected areas decreased to 2541.3 ha, followed by further reductions in 2021 (~ 1362.4 ha) and 2022 (~ 1380.7 ha), suggesting partial canopy stabilization and ecosystem recovery. From 2023 to 2024, affected areas slightly increased to 2198.5 ha and 2065.3 ha, respectively, indicating continued vulnerability despite lower outbreak intensity. Analysis of repeated vegetation changes over seven years showed that only a small fraction of the area experienced recurring changes, highlighting the spatial heterogeneity of oak leaf-roller impacts. The expansion of affected areas in 2019 demonstrates a lagged response to the 2017 extreme drought. This suggests that physiological weakening from severe moisture deficits (low SPEI) creates a window of vulnerability that defoliating pests exploit in subsequent years^38^. This synergy between climatic pulses (SPEI) and biotic shocks (BFAST breakpoints) offers a more nuanced framework for understanding Zagros forest decline.

Chi-square analyses confirmed that outbreak patches were non-randomly distributed across topographic variables. The highest density occurred at mid-elevations (1200–1800 m), on steep slopes (> 30%), and on north-facing aspects. Patches were concentrated within 0–500 m of roads and beyond 500 m from rivers, indicating that both natural and anthropogenic factors influence pest distribution. High infestation density near roads likely stems from synergistic factors: dust-induced physiological stress, anthropogenic pest transport, and edge effects that create microclimates favorable for *T. viridana* colonization^39^. Conversely, the scarcity of outbreaks within 500 m of rivers suggests that riparian moisture enhances tree vigor and chemical defenses, reducing pest success. Beyond this threshold, increased moisture stress likely compromises host resistance, facilitating more extensive defoliation^40^.

Ecologically, these results suggest that forest decline in the Zagros is driven directly by bud consumption and reduced tree photosynthetic capacity, and indirectly by synergistic effects of drought and climate variability. This interpretation is supported by Dargahian, et al.^41^, who documented a notable coincidence between drought events and oak forest decline. Analysis of NDWI values and reduced tree moisture indicates that weakened tree physiology creates favorable conditions for pest population expansion, leading to recurrent outbreak cycles and cumulative damage. Unlike the gradual and widespread impact of droughts, the *T. viridana* outbreaks detected here exhibited a distinctive rapid defoliation-refoliation cycle within the larval activity window, allowing for the differentiation of biotic stress from climatic signals^42^.

BFAST outputs revealed a distinct spatial pattern of canopy decline. The densest patches corresponded to areas with previous infestations and gradually spread to lower slopes and peripheral zones. These findings align with Lambert, et al.^25^, confirming BFAST’s capability to detect both abrupt and gradual vegetation changes induced by pest outbreaks in long-term time series. Crucially, the absence of near-zero vegetation index values and visible burn scars in high-resolution imagery (Google Earth/ESRI) rules out wildfires as the cause of the 2019 breakpoints.

From a management perspective, monitoring efforts should prioritize high-risk areas, mid-elevation zones and moderately sloped terrains exhibiting the highest pest sensitivity. Integrating Sentinel-2 imagery with BFAST and vegetation indices can serve as an effective early-warning system. Based on the findings of this study, preventive ecological measures, such as watershed management, soil moisture enhancement, and selective pruning, are recommended to strengthen tree resilience. Integrated Pest Management (IPM) strategies, including biological control and grazing reduction, are essential to mitigate the impacts of oak leaf-roller outbreaks on forest health.

### Study limitations and future perspectives

Several limitations should be acknowledged. First, the 10–20 m spatial resolution of Sentinel-2 precludes detection at the individual branch or leaf scale. Second, persistent cloud cover in the Zagros region occasionally created gaps in time-series continuity. Furthermore, while field data were limited to 2022–2024, validation of earlier outbreaks (e.g., 2019) relied on historical imagery and official records.

Reduced data availability during early spring, primarily due to cloud cover, represents an additional constraint for detecting short-term spectral anomalies during the larval feeding window (late spring). Sparse observations may obscure or attenuate abrupt declines in vegetation indices, potentially delaying or weakening breakpoint detection. Consequently, the magnitude and timing of the observed spectral declines, interpreted as a decoupling from the regional greenness trend, should be considered conservative estimates of true disturbance intensity.

Regarding spectral analysis, the structural heterogeneity of oak stands influences absolute vegetation index values. Some affected stands exhibited lower baseline values even prior to breakpoint detection, likely reflecting pre-existing structural differences or historical disturbance legacies rather than solely immediate outbreak effects. Since these factors cannot be fully quantified from available remote sensing data, they introduce uncertainty in attributing all changes exclusively to the analyzed pest events. While the BFAST algorithm emphasizes relative temporal anomalies, these baseline differences may still influence the magnitude and trajectory of detected changes. Furthermore, distinguishing between biotic and abiotic stressors remains a fundamental limitation; delayed leaf onset or variations in leaf water content can produce signals similar to defoliation. Although integrating high-frequency observations with multi-year field data reduces this ambiguity, future studies incorporating hyperspectral imagery or in-situ physiological measurements would further enhance the separation of these interrelated effects.

Finally, while Chi-square identified key spatial associations, future research using multivariate approaches (e.g., Random Forest) could better decouple the inter-correlated effects of elevation and slope. Nevertheless, the integration of Sentinel-2 imagery, BFAST, and vegetation index analysis provides a coherent and data-driven representation of the spatiotemporal patterns associated with pest-related disturbances in the Zagros. These findings establish a solid scientific and practical foundation for designing smart management policies and preventive interventions. Specifically, this approach supports the development of early-warning systems that utilize Sentinel-2 time-series anomalies to prioritize zones with emerging stress signals, ultimately enhancing the resilience of Zagros forests against escalating biotic and climatic crises.

## Conclusion

This study addressed three key research questions concerning the capability of Sentinel-2 imagery, the performance of the BFAST model, and the influence of topography on pest infestation patterns. Our results show that outbreaks of the green oak leaf-roller (*T. viridana* L.) are one of the major drivers of reduced forest vitality in the oak forests of Sardasht and Piranshahr counties of southern West Azarbaijan Province. High-resolution Sentinel-2 data allowed precise detection of vegetation changes related to pest activity, effectively distinguishing declining stands from healthy ones and revealing localized canopy degradation often missed by coarser datasets. Among the indices examined, NDVI and EVI were highly sensitive to reductions in canopy vigor and photosynthetic performance, while NDWI effectively captured variations in canopy moisture and drought-induced stress.

Time-series analysis revealed that the intensity of vegetation changes between 2018 and 2024 was primarily driven by cyclical pest outbreaks of the *T. viridana*, with drought acting as a secondary modulating factor, The year 2021 marked the peak of this combined stress. Topographic factors significantly shaped spatial patterns of vegetation loss, with mid-elevations (1200–1800 m) and moderately sloped terrains (15–30%) exhibiting the highest susceptibility. These findings highlight that microclimatic conditions and forest structure, modulated by elevation and slope, are critical determinants of pest impact and canopy vulnerability, providing actionable insights for targeted forest management. BFAST analysis identified the largest area of significant vegetation change (~ 4253 ha) in 2019, coinciding with peak *T. viridana* density, followed by a decrease to ~ 1362 ha in 2021, suggesting partial stabilization. These findings confirm that spectral indices and time-series models complement each other effectively in quantifying canopy dynamics and detecting pest outbreak cycles.

From a management perspective, integrating satellite-based monitoring, vegetation indices, and time-series modeling provides a robust framework for early detection and adaptive management. Prioritizing mid-elevation, moderately sloped areas, alongside drone-based monitoring, early-warning systems, and Integrated Pest Management strategies, such as biological control and soil moisture enhancement, can strengthen the resilience of Zagros oak forests to both biotic and climatic stressors.

## Methods

### Study area

**Fig. 8:**
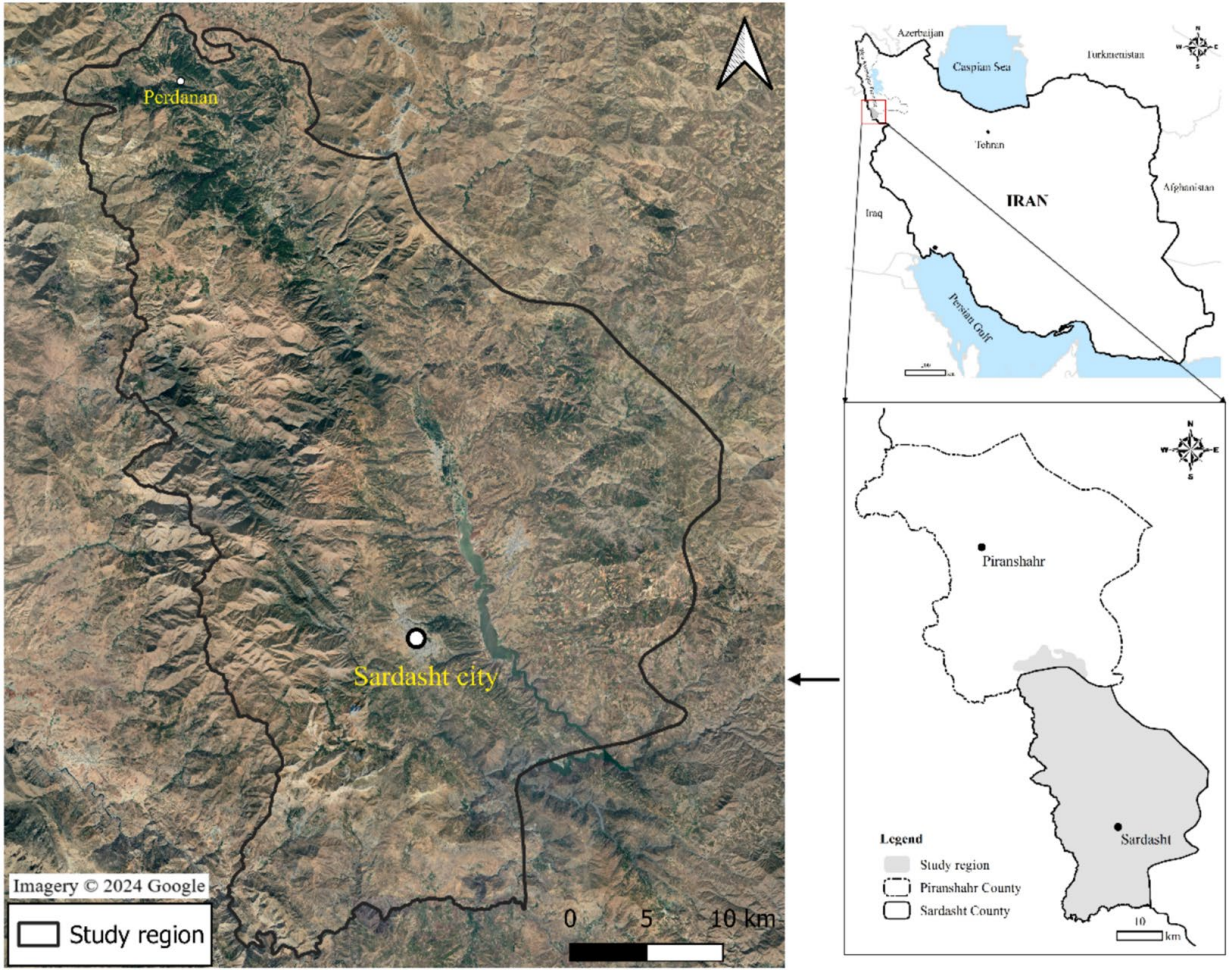
Location of the study area (NW Iran). Background: Google Satellite imagery. The map was prepared using ArcMap 10.8.2 (Esri, https://www.esri.com) and QGIS 3.40.7 (QGIS Development Team, https://qgis.org).

The study area is located in northwestern Iran, within West Azerbaijan Province, encompassing the entire Sardasht County and a very small portion of Piranshahr County, known as the Perdanan forests (Fig. 8). This region marks the northern extent of the Zagros forest belt and is geographically situated between 45°17′ to 45°19′ E longitude and 35°28′ to 36°30′ N latitude. Mean elevations are approximately 1360 m in Piranshahr and 1510 m in Sardasht, with general slopes ranging between 30 and 40%. Mountainous topography interspersed with valleys and limited plains creates a unique physiographic landscape, and the forested area in Sardasht county was estimated at 64,887 ha in 2023^43^. The climate exhibits a warm and humid regime in Piranshahr and cold and humid conditions in Sardasht, influenced by Black Sea and Mediterranean air masses. Mean annual precipitation in Perdanan forests during 2005–2020 was approximately 692 mm with an average annual temperature of 13.8 °C, whereas Sardasht records up to 950 mm annual rainfall. Notably, despite these regional variations, the microclimate of the specific study area within the Perdanan forests is more representative of Sardasht’s cold and humid regime than that of Piranshahr, providing the consistent environmental conditions necessary for this study. The forests of this region represent part of the ecologically valuable Northern Zagros ecosystem, dominated by *Quercus brantii*, *Q. libani*, and *Q. infectoria*. Other important tree species include *Pistacia atlantica*, *Prunus scoparia*, *Acer monspessulanum*, *Crataegus* spp., *Juglans regia*, *Ficus carica*, *Salix* spp., and *Populus* spp.

### Satellite data

#### Primary data source

The main data source for this study consisted of Sentinel-2 Level-2A Surface Reflectance Harmonized imagery provided by the European Space Agency^44^. Sentinel-2 images were retrieved for the period spanning 2018 to 2024. For each year within this time series, data selection focused on the window from late March to late October (specifically from 21 March to 22 October). This seasonal range corresponds to the active life cycle of the oak leaf-roller moth (*T. viridana*), which is univoltine (having a single generation per year), and covers the full progression from initial larval feeding to the end of the growing season. This period was chosen to cover the entire activity of larvae, from emergence to pupation, and to capture the resulting canopy responses. These data are preprocessed to surface reflectance with atmospheric corrections applied, removing effects such as molecular scattering and water vapor absorption, thus making them suitable for vegetation index calculations^45^. Sentinel-2 imagery is widely used in forestry studies for canopy health assessment and monitoring forest responses to biotic and abiotic stressors.

Sentinel-2A and 2B satellites, in sun-synchronous orbit, provide data with a five-day revisit cycle^46^, enabling detailed monitoring of canopy dynamics, including forest pest outbreaks such as the oak leaf-roller moth. Spatial resolution varies by spectral band from 10 to 20 m; visible and near-infrared (NIR) bands at 10 m are optimal for assessing leaf area and detecting defoliation patches, while shortwave infrared (SWIR) bands at 20 m facilitate monitoring canopy and soil moisture, critical for evaluating forest health under drought or pest stress.

Visible bands (blue, green, red) provide direct information on plant pigments, particularly chlorophyll, and are useful for detecting physiological stress and vegetation decline^47^. NIR reflects leaf internal structure and canopy density, serving as a key indicator of biomass and vegetation vigor^48^. SWIR bands detect water content variations in leaves and soil. Combining these bands allows calculation of indices such as NDVI, EVI, and NDWI, widely applied for forest health monitoring and discriminating healthy versus stressed trees^49^.

All Sentinel-2 data were accessed and processed via the Google Earth Engine (GEE) cloud computing platform, which provides an extensive archive and high computational capacity, enabling efficient time-series analysis and implementation of change detection algorithms over large forested areas in West Azerbaijan (Piranshahr and Sardasht). The platform facilitated scalable preprocessing, vegetation index computation, and extraction of dense temporal observations required for subsequent analysis. The complete GEE implementation of the processing workflow used in this study is publicly available in a GitHub repository, with an archived version accessible via Zenodo (10.5281/zenodo.19347108), ensuring full reproducibility of the analysis.

#### Preprocessing

A critical step in remote sensing analysis is precise data preprocessing^50^. To ensure that detected canopy changes were not artifacts of cloud contamination, a robust multi-stage masking pipeline was implemented in Google Earth Engine (GEE). Instead of relying solely on the QA60 bit-mask, which can under-detect thin cirrus and cloud edges, we primarily employed the S2cloudless probability collection (with a 30% threshold)^51^ and the Cloud Score + algorithm^52^. These machine-learning-based methods provided superior discrimination between cloud-contaminated pixels and true vegetation signals. For each image in the 2017–2024 time series, we applied a combined mask: pixels identified as clouds by S2cloudless or characterized by low quality scores in Cloud Score + were excluded. Furthermore, a 200-m buffer was applied around detected cloud masks to eliminate shadow-affected edges. These rigorous preprocessing steps ensured that the subtle canopy dynamics analyzed via BFAST were derived from high-quality, cloud-free observations.

Spatial clipping was applied based on the study area to reduce data volume and focus the analysis on the Zagros forests in southern West Azerbaijan.

#### Ancillary data

For comprehensive analysis, a 12.5 m resolution Digital Elevation Model (DEM) and administrative boundary maps of counties and forests were used. The DEM facilitated extraction of topographic variables (slope, aspect, elevation) which influence microclimatic conditions and pest distribution^53^. Boundary maps ensured precise overlay of spectral and spatial layers, critical for generating accurate pest distribution maps.

### Vegetation index calculation

NDVI, EVI, and NDWI were used to monitor oak forest health. These indices are highly sensitive to vegetation biomass and physiological condition and are widely applied in forestry and pest monitoring studies^54^.

NDVI (Normalized Difference Vegetation Index):

1 $$NDVI=\frac{NIR-Red}{NIR+Red}$$

Values range from − 1 to + 1; higher values indicate healthy, dense vegetation, while values near zero or negative correspond to bare soil or water^55^. NDVI is effective for detecting pest-affected areas and seasonal vegetation changes.

EVI (Enhanced Vegetation Index):

2 $$EVI=2.5\times \frac{NIR-Red}{NIR+6\times Red-7.5\times Blue+1}$$

EVI reduces saturation effects in dense vegetation and mitigates atmospheric interference, providing more precise discrimination of healthy and stressed vegetation compared to NDVI^56^. EVI values range from − 1 to + 1, where values closer to + 1 represent dense and healthy forest canopies, while values approaching 0 or below indicate vegetation stress, defoliation, or non-vegetated surfaces^57^.

NDWI (Normalized Difference Water Index):

3 $$NDWI=\frac{Green-NIR}{Green+NIR}$$

Positive values indicate surface water, negative values correspond to dry soil or vegetation. NDWI allows monitoring of drought impacts and indirect pest effects^58^.

Combining NDVI, EVI, and NDWI enables multidimensional analysis of forest condition, identification of sensitive areas, and supports sustainable forest management planning.

### Drought index calculation (SPEI)

To assess the climatic drivers of forest disturbance and their correlation with pest outbreaks, the Standardized Precipitation Evapotranspiration Index (SPEI) was utilized^59^. The SPEI is a multi-scalar drought index that accounts for both precipitation and potential evapotranspiration (PET), making it particularly effective for monitoring drought-induced stress in forest ecosystems under warming conditions.

In this study, the 3-month SPEI (SPEI-3) was calculated for the Sardasht region, covering a long-term period from 1996 to 2024. The 3-month scale was selected to capture short-term to intermediate seasonal moisture deficits that directly influence the physiological vigor of oak trees and the life cycle of *T. viridana*. The calculation was performed using the '*SPEI*' package in R software^60^, employing the Thornthwaite equation to estimate PET based on monthly temperature and precipitation data.

The calculated SPEI values were interpreted based on the standard drought classification^61^, where values strictly within ± 0.99 denote near-normal conditions. Values ranging from − 1.0 to − 1.49, − 1.5 to − 1.99, and ≤  − 2.0 signify moderate, severe, and extreme drought, respectively. Conversely, positive values between 1.0 and 1.99 indicate moderate to very wet conditions, while values ≥ 2.0 represent extremely wet periods.

### Vegetation change detection using BFAST

BFAST (Breaks For Additive Seasonal and Trend) is an advanced framework for time-series analysis of vegetation indices, capable of detecting both abrupt and gradual changes simultaneously^62^. It is widely applied in forestry and ecology for monitoring pest outbreaks, drought, wildfire, and land-use change^63^. BFAST decomposes time-series data into long-term trend, seasonal pattern, and residual components, enabling precise identification of breakpoints corresponding to significant ecological events, such as pest infestations affecting canopy health.

### Mathematical model

The additive model underlying BFAST decomposes vegetation index time series as follows^64^:

4 $${Y}_{t}={T}_{t}+{S}_{t}+{e}_{t}$$

where *Y*_*t*_ is the observed vegetation index (NDVI, EVI, or NDWI) at time t; *T*_*t*_ represents the long-term trend; *S*_*t*_ is the seasonal component; and *e*_*t*_ is the remainder, encompassing random variation, sensor noise, and non-systematic environmental effects. Breakpoints derived from piecewise regression indicate significant vegetation changes attributable to pests or environmental stressors^65^.

### Implementation in the current study

BFAST was systematically applied to daily time-series of NDVI, EVI, and NDWI derived from Sentinel-2 imagery, spanning 2018–2024. This framework intrinsically detects abrupt and gradual changes by modeling the seasonal component over the entire time series, allowing for the differentiation of normal phenological cycles from stress-induced deviations based on signal-to-noise ratios^26^.

The BFAST algorithm was implemented on a pixel-based time-series framework derived from Sentinel-2 imagery (2018–2024). Following stringent cloud masking and quality filtering, the Sentinel-2 time series (2018–2024) maintained a high observational frequency. Across the study period, the dataset yielded a total average of approximately 405 valid observations per pixel. On an annual basis, the number of clear-sky observations ranged from a minimum of 27 to a maximum of 134, with a consistent mean of 56–60 observations per year (Table 4). This dense temporal sampling provided a robust foundation for the BFAST algorithm to characterize the stable seasonal component and define each pixel’s historical baseline with high statistical confidence^20^. Baseline phenological conditions were characterized individually for each pixel, utilizing its intrinsic historical seasonal trajectory rather than static spatial references. Notably, data sparsity was more pronounced during early spring due to persistent cloud cover and topographic snow masking, which constrained the observational frequency during initial canopy development. To ensure spatiotemporal continuity, linear interpolation was integrated within the BFAST framework; however, this reconstruction approach may impose a smoothing effect on the abrupt spectral fluctuations associated with the onset of larval defoliation^66^. Consequently, breakpoints detected during this critical phenological window should be interpreted with caution, as the spectral magnitude and temporal precision may represent conservative estimates (i.e., potential underestimation) of the true disturbance intensity.

**Table 4:** Summary of Sentinel-2 time-series observation density after cloud masking and quality filtering (2018–2024).

Year	Minimum Observations	Maximum Observations	Mean Observations
2018	29	120	56.03
2019	32	131	60.01
2020	32	126	58.94
2021	31	131	60.38
2022	32	134	60.50
2023	27	114	52.63
2024	32	124	56.84
Total	215	880	** ~ **405

A key advantage of this approach is its focus on temporal anomalies relative to each pixel’s own historical baseline, rather than absolute vegetation index values which are often influenced by varying forest stand density^31^. This process effectively normalizes the influence of canopy cover and regional climatic gradients, as breakpoints are identified based on abrupt deviations from a stable phenological trend^20^. Specifically, stands were categorized into the affected class if the model identified a significant structural breakpoint (*p* < 0.05) during the peak larval feeding window (April–June) that resulted in a negative magnitude of change. Conversely, stands that exhibited a stable seasonal component without significant breakpoints, or those showing only minor fluctuations within the range of natural variability, were grouped as healthy stands^67^.

To ensure data reliability, preprocessing included atmospheric correction, cloud/shadow removal, and spatial clipping to the study area. Preprocessing also included linear interpolation to maintain continuity during early spring gaps (see above). Finally, BFAST extracted Trend, Seasonal, and Remainder components to determine the timing, magnitude, and spatial location of vegetation changes, providing a robust method for mapping oak leaf-roller moth infestation patches and monitoring canopy health dynamics independent of the inherent environmental differences between the administrative regions.

**Fig. 9:**
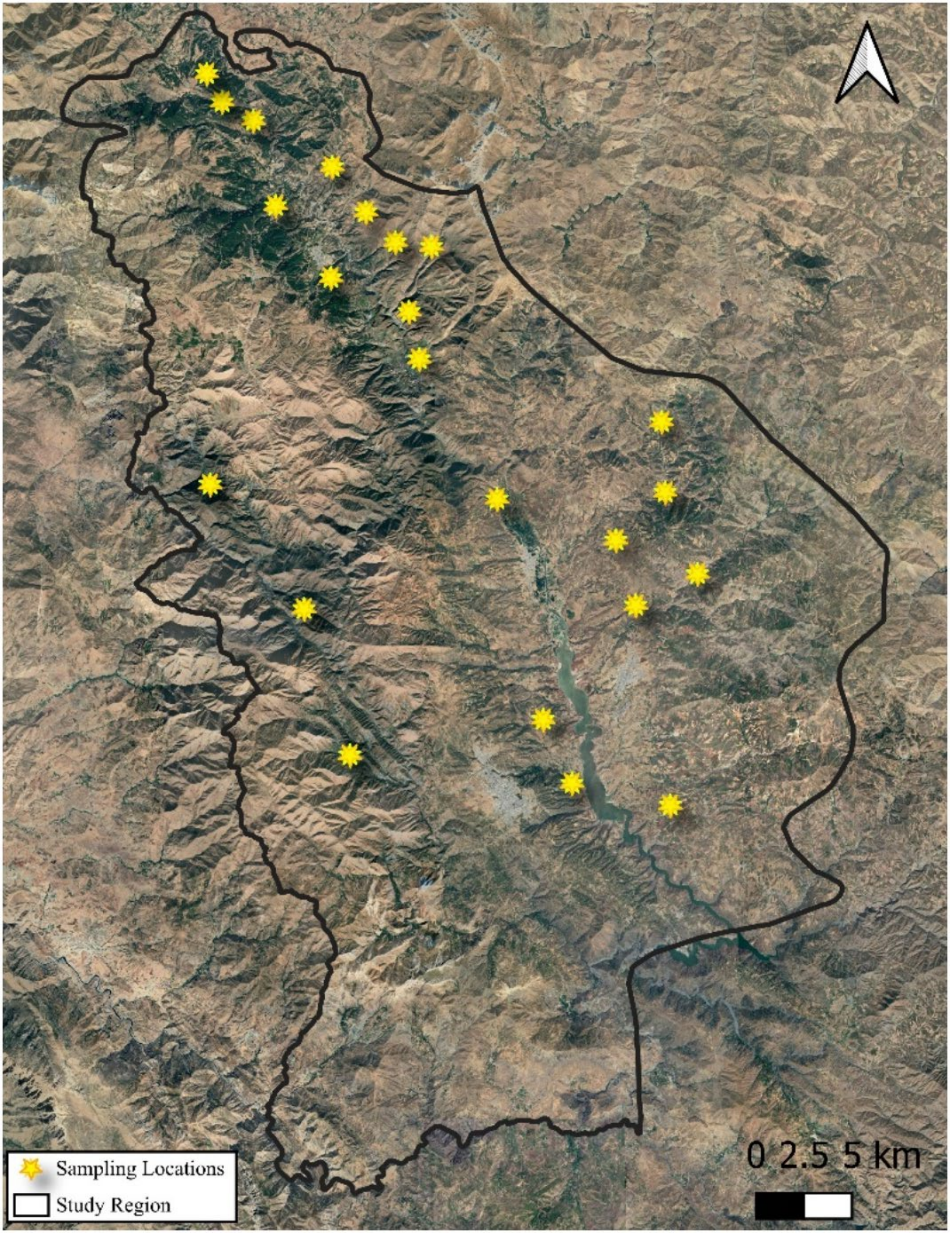
Spatial distribution of the field reference points and sampling locations across the study area. The map was prepared using QGIS 3.40.7 (QGIS Development Team, https://qgis.org).

Validation was conducted using 100 field reference points sampled across 24 distinct locations (spatial distribution shown in Fig. 9). Field data collection was strategically timed to coincide with peak larval activity and maximum defoliation periods (late April–June) in 2022, 2023, and 2024. During these surveys, defoliation was confirmed through direct visual inspection of the canopy and the presence of *T. viridana* larvae, establishing a direct link between biotic stress and spectral anomalies.

For the 2018–2021 period, retrospective validation was performed by cross-referencing high-resolution historical satellite imagery from Google Earth Pro and Google/ESRI databases within a QGIS environment. This visual assessment, supplemented by multi-year NDVI profile analysis, verified the timing and magnitude of the detected breakpoints. The performance of the BFAST-detected changes was then quantitatively evaluated through confusion matrices. Specifically, Overall Accuracy (OA), Kappa coefficient, Producer’s Accuracy, User’s Accuracy, as well as Commission and Omission errors, were calculated based on a pixel-to-pixel comparison, where the classification of each of the 100 reference points was matched against the corresponding BFAST algorithm output for each study year.

### Environmental and anthropogenic variables

To examine the drivers of forest disturbance, five environmental and anthropogenic factors were selected: elevation, slope, aspect, distance to roads, and distance to rivers. Elevation was categorized into three zones (< 1200 m, 1200–1800 m, and > 1800 m), while slope was classified into low (0–15%), medium (15–30%), and steep (> 30%) classes. Aspect was divided into five categories (North, South, East, West, and Flat). Anthropogenic pressures were represented by distance to roads (0–500 m, 500–1000 m, > 1000 m) and distance to rivers (0–200 m, 200–500 m, > 500 m). These factors were chosen due to their influence on microclimate, pest dynamics, and human-induced forest degradation.

### Statistical analysis

Statistical analyses were performed to evaluate the spatial distribution of forest decline across environmental and anthropogenic gradients. Since the field data focused on confirmed infested locations, a Chi-square (chi^2^) Goodness-of-Fit test was employed to determine if the observed frequency of decline points significantly deviated from an expected uniform distribution across different classes of elevation, slope, aspect, and proximity factors. This analysis identifies whether forest vulnerability is disproportionately higher in specific environmental conditions. All analyses were conducted in SPSS 27 at a 95% confidence level (α = 0.05).

## Data Availability

The Sentinel-2 Level-2A surface reflectance data used in this study are publicly available through the Google Earth Engine (GEE) platform. The GEE processing workflow, including cloud masking (S2cloudless; threshold < 30% with a 200 m buffer), vegetation index calculation (NDVI, EVI, NDWI), and time-series extraction, is openly available in the following public repository: https://github.com/HadiBey-Ourm/OakLeafRoller_GEE- A permanently archived version of the repository is available on Zenodo at https://zenodo.org/records/19347108 (10.5281/zenodo.19347108). The BFAST breakpoint detection analysis was performed externally in R/Python using the exported time-series data. Additional data supporting the findings of this study are available from the corresponding author upon reasonable request.
